# Policy solutions to improve access to fertility treatment and optimise patient care: consensus from an expert forum

**DOI:** 10.3389/frph.2025.1605480

**Published:** 2025-08-29

**Authors:** G. David Adamson, Hannah Armstrong, Ying Cheong, Elaine Damato, Human Fatemi, Rui Ferriani, Georg Griesinger, William Leigh Ledger, Michele Pistollato, Antonio Pellicer, Angelina Petrova, Luk Rombauts, Tim Wilsdon, Søren Ziebe

**Affiliations:** ^1^ International Committee for Monitoring Assisted Reproductive Technologies, Vancouver, BC, Canada; ^2^ Equal3 Fertility, Cupertino, CA, United States; ^3^ Charles River Associates International, Inc., London, United Kingdom; ^4^ University of Southampton, Human Development and Health, Southampton, Hampshire, United Kingdom; ^5^ ART Fertility Clinics LLC, Abu Dhabi, United Arab Emirates; ^6^ University of São Paulo, Ribeirão Preto, Ribeirão Preto, São Paulo, Brazil; ^7^ Member of Camera Technique of Regional Medicine Council, São Paulo, Brazil; ^8^ Department of Gynaecological Endocrinology and Reproductive Medicine, University Hospital of Schleswig-Holstein, Lübeck, Germany; ^9^ School of Women's and Children's Health, University of New South Wales, Kensington, NSW, Australia; ^10^ Fertility and Research Centre, Royal Hospital for Women, Randwick, NSW, Australia; ^11^ IVIRMA Valencia, Valencia, Spain; ^12^ Monash IVF, Cremone, NSW, Australia; ^13^ Department of Obstetrics & Gynaecology, Monash University, Clayton, VIC, Australia; ^14^ Laboratory of Reproductive Biology, The Fertility Department, Rigshospitalet, University Hospital of Copenhagen, Copenhagen, Denmark

**Keywords:** fertility treatment, fertility policy, infertility, policy recommendations, assisted reproductive technology

## Abstract

**Background:**

Infertility is an underrecognized disease which affects over 17% of the reproductive age population worldwide. However, availability of, and access to, assisted reproductive technology (ART) is variable across countries. There are significant challenges relating to awareness, financial and other barriers to care, cultural considerations, and the level of support provided to people undergoing care. Previous studies have explored these challenges, but less attention has been given to the policy implications. As the need for fertility care rises, we investigate the evidence that policy changes can be implemented to improve access to ART treatment.

**Methods:**

A review of literature was conducted on fertility policy challenges and developments, covering fertility recognition and awareness; cultural and religious considerations; and access to ART treatment, psycho-social care, and supplementary care. Nine medical and academic experts were invited to validate secondary research findings and provide their perspectives on policy implications. The experts covered different specialties and geographic expertise. Experts participated in individual 60-minute interviews, then a half-day Policy Forum discussion was held virtually in May 2023.

**Results:**

Lack of recognition of infertility as a disease, low financial coverage of fertility services, limited psychosocial support, and cultural considerations are substantial barriers to fertility services access. Some countries have limited reimbursement of services or offer only private care, significantly limiting treatment access. Others restrict reimbursement based on age, gender and family status, which creates access inequities. Policy action is needed to mitigate these challenges and to ensure timely and equitable access to fertility care. Decision-makers need to collectively recognize infertility as a disease, rather than just a social issue. Equity of access to infertility services should be ensured by expanding the availability of public funding, along with review and rationalisation of criteria for treatment reimbursement. To improve engagement in treatment and support through the fertility journey, access to psychosocial care should be expanded and included as a core service.

**Conclusion:**

Major obstacles to accessing ART treatment have been identified across regions globally, highlighting the urgent need for national policy action to enhance care quality by reviewing current legislation, improving patient and physician education, refining reimbursement procedures, and expanding psychosocial support services.

## Introduction

Globally, an estimated one of every six people is affected by the inability to have a child (1, 2). Infertility prevalence estimates are similar across countries with different income levels, but they differ across geographies, with lifetime infertility ranging from 10.7% in the World Health Organization (WHO) Eastern Mediterranean Region to 23.5% in the WHO Western Pacific Region (1). Infertility is one of the factors (in addition to socioeconomic pressures) fuelling the difference between the number of children that people would like to have and the final fertility rate across most countries: a study focused on Europe and the United States (US) found that actual fertility was always below the mean intended family size measured in young adulthood (3). As most evidence and policy discussions centre around infertility of women, this paper will primarily focus on female infertility. Additionally, most treatment options focus on treating women. We would like to emphasise that men also suffer from infertility and more understanding of managing this disease is needed; however, this is outside the scope of this paper. Further, reproductive diseases in one partner may lead to infertility for both partners.

Involuntary childlessness can have devastating social and psychological impacts, such as ostracism, anxiety, depression, and low self-esteem (4). Studies have found that women^1^ affected by primary infertility i.e., those who have never been pregnant, show a higher prevalence of depression and anxiety than women with no infertility issues (5). Particularly in low- or middle-income countries (LMICs), the consequences of infertility are significant due to the stigma and associated cultural implications; one in three women experiencing infertility in LMICs suffer from intimate partner violence each year (4, 6). High-income countries (HICs) are not exempt from the impact of cultural stigma surrounding infertility and fertility treatment, as the impact is often associated with a country's cultural context rather than income level. A Japanese study found that women undergoing fertility treatment experienced harassment in the workplace and were not provided with the necessary support, resulting in one-sixth of women resigning after starting treatment (7).

Simultaneously, total fertility rates (TFR) are gradually declining; in 2017 the TFR was just above replacement level (traditionally defined as 2.1 children per woman) globally (8, 9). Projections indicate that 23 nations—including Italy, Japan, Spain, and South Korea,—will see their populations halve by 2100 (10). This will trigger unprecedented socio-economic change, including gaps in collected taxes—which may impact pensions and healthcare provisions for the retired—and a limited workforce to support economic growth and provide aged-care (11). While trends in voluntary childlessness may also impact these projections, infertility is a medical condition requiring access to adequate treatment and care which may be contributing to the decline in TFR.

Despite male factors playing a significant role in a couple's infertility—with some studies citing that 40%–50% of infertility cases are attributable to male factor infertility—the treatment burden falls mainly on women and therefore, will be the focus of this paper (12, 13). Approximately 30% of female infertility cases are classified as “unexplained” due to a lack of an obvious cause (14). After this, the most common reasons for infertility in women are ovulation problems, endometriosis, poor egg quality, polycystic ovarian syndrome (PCOS), and fallopian tube problems (often resulting from untreated sexually transmitted infections) (15); however, the relative proportion of diagnoses associated with infertility varies significantly globally. Treatment options can be summarised as treatment with drugs to help with ovulation (16), intrauterine insemination (IUI), surgery or ART such as *in vitro* fertilization (IVF). Services such as egg freezing for medical or other reasons can potentially support further fertility preservation.

Since 2009, the WHO has recognised infertility as a disease and stated that to address infertility effectively, health policies need to acknowledge that it can often be treated (13). This led to the inclusion of infertility in the International Classification of Diseases (ICD) (17). Nevertheless, many governments still do not perceive infertility as a disease, so it is not deemed to be a medically necessary covered benefit by some public and private healthcare providers (17).

Reflecting the growing burden of infertility, demand for ART treatments is growing globally. The International Committee for Monitoring ART (ICMART) (18) has reported that ART utilization (expressed as the number of IVF cycles) increased by 13.3% between 2017 and 2018 across 79 countries (19). By 2025, it is estimated that approximately 15 million babies have been born as a result of ART (20). Despite this growing global trend, there are still notable discrepancies in the access and availability of ART treatment globally. Economic factors are the chief contributors to disparities in access to effective treatment; however, geographic, social and cultural factors, including individual or systemic discrimination that disadvantages certain people because of their race, ethnicity, sexual orientation, marital status or gender identity play a role as well (21). ART remains underfunded, resulting in limited public reimbursement, making it inaccessible to many due to the high out-of-pocket costs and limited availability of publicly funded ART centres (13). Although many studies have demonstrated cost-effectiveness of IVF and the return on investment in IVF (22, 23), public coverage is limited which creates an affordability challenge especially for patients in LMICs and LICs (6, 24). Consequently, the high out-of-pocket cost impacts the utilization of ART services, leading to low uptake. For example, in Japan and Australia, women from lower-income households seek less medical help for their infertility (25, 26).

Some countries have implemented national plans covering infertility, typically as part of broader women's health strategies that aim to improve access to fertility treatments; the Women's Health Strategy for England is a ten-year strategy to improve women's health, and one of its pillars focuses on improving infertility education and care (27). Similarly, the Australian National Women's Health Strategy aims to promote awareness of infertility and to strengthen access pathways to sexual and reproductive health services (28). However, in most countries, infertility policies and services are broadly considered inadequate, with healthcare professionals and academics advocating for the introduction of national plans or more comprehensive infertility policies (2, 29).

This suggests a need for global action to address infertility, which would help achieve the health and gender-equality targets of the 2030 UN Sustainable Development Goals, which advocate for universal access to sexual and reproductive health and reproductive rights for women (30). The WHO is advocating for widening access to fertility care and making it a priority for health researchers and policymakers so that safe, effective, and affordable ways to attain parenthood are available to all (13). This paper aims to identify the existing policy barriers that prevent or impede infertility patients' ability to access optimal care and to propose policy recommendations to address these barriers.

## Methods

The findings of this paper derive from a three-step approach culminating in an expert Policy Forum. We reviewed the recent literature, published between 2002 and 2024, on fertility policy developments, associated policy gaps and challenges, and examples of successful policy implementation. We searched for publications focusing on the following topics: infertility recognition and awareness; cultural and religious considerations around fertility; and access to ART treatment, psychosocial care, and supplementary care. The literature review covered global studies and publications without a specific country focus. To find relevant publications, the search terms “assisted reproductive technology”, “fertility policy”, and “challenges” or “best practices” were used in Google Scholar and PubMed. The retrieved publications were ranked according to their relevance to the search terms, along with additional articles located through a targeted search. A total of 130 articles were reviewed.

In the second step, one-to-one interviews were scheduled with nine fertility experts (Table 1). The experts were selected based on their expertise to ensure the sample covers large multinational fertility clinics, academic fertility research groups, and involvement in medical societies. Additionally, the experts were selected based on their country of practice to ensure the sample includes expertise from different geographic regions, levels of economic development, and TFRs. We obtained feedback on the barriers to ART access and care and discussed possible best practices in policymaking to support optimal access and care.

**Table 1:** List of experts who participated in the one-to-one interviews.

Name	Country of residence	Affiliation
Prof. G. David Adamson	United States	Clinical Professor ACF, at Stanford University School of Medicine and Associate Clinical Professor at University of California San Francisco School of Medicine
Prof. Ying Cheong	United Kingdom	Professor of Reproductive Medicine, University of Southampton
Prof. Human Fatemi	United Arab Emirates	Medical Director of ART Fertility Clinics
Prof. Rui Ferriani	Brazil	Professor Obstetrics Gynaecology, University of São Paulo
Prof. Georg Griesinger	Germany	Professor of Gynaecological Endocrinology and Reproductive Medicine at Luebeck University
Prof. William Ledger	Australia	Head and Professor of Discipline of Women's Health, School of Clinical Medicine, University of New South Wales Director of Reproductive Medicine, Royal Hospital for Women, Sydney
Prof. Antonio Pellicer	Italy	IVIRMA (global medical reproductive institution), Executive Chair
Prof. Luk Rombauts	Australia	Adjunct Clinical Professor in the Department of Obstetrics and Gynaecology at Monash University Head of Reproductive Medicine at Monash Health, Southern Health
Prof. Søren Ziebe	Denmark	Head of the Fertility Department, Juliane Marie Centre—Rigshospitalet, Copenhagen University Hospital, Denmark

The final step was to convene a virtual Policy Forum of the above experts, facilitated by Michele Pistollato, Hannah Armstrong, Elaine Damato, and Angelina Petrova on 24 May 2023. During the Policy Forum, in addition to reaching a consensus on the barriers to ART access and identifying existing best practices, the experts discussed the factors a fertility policy needs to be successful and co-developed implementable and actionable policy goals to support optimal patient access and care. Additional one-to-one geographical tailoring sessions were conducted with two of the experts to obtain regional perspectives on the policy recommendations.

## Challenges in accessing ART treatment and fertility care

We have segmented challenges into five categories as a tool to help us discuss them and to identify country-specific access barriers.

• Recognition and awareness
• Access to ART treatment
• Psychosocial support
• Other supplementary care
• Cultural, social, and religious considerations

To analyse the extent of the problem, we will describe the specifics of each challenge and how it can manifest at a country level (based on input from the expert Policy Forum) and review evidence of how it impacts patient access to treatment.

### Recognition and awareness

According to the experts from the Policy Forum, there are widespread limitations in the extent of recognition of infertility as a disease (31). The WHO has classified infertility as a serious disease; however, relative to other diseases it has not been given priority by national policymakers, resulting in decreased investment into research initiatives, preventative programmes, and treatment (32). Countries across the globe can be categorised into three archetypes concerning their approach to recognising infertility as a disease. Some countries are closely aligned with the WHO and have recognised infertility as a disease, resulting in the introduction of mechanisms to enable access to ART treatment. A pioneering example of this is Australia, or specifically the Australian State of Victoria, which was the first jurisdiction in the world to pass extensive legislation to regulate the use of ART in 1984 (33). Other countries have more recently recognised infertility as a disease but are yet to establish dedicated policies and consistent funding. For example, while clinics in Poland have been offering ART treatment since the 1980s, funding for such procedures was not introduced until 2013, and then halted in 2016 due to a change of government and policy (34). Lastly, a selection of countries have not yet invested into addressing infertility and providing associated care, often due to competing priorities. For instance, countries with high TFRs may be more focused on family planning initiatives such as contraception provision, rather than establishing infertility policies (32).

#### Lack of awareness of relationship between age and fertility

Fertility rates in most countries continue to fall, with Europe demonstrating the lowest regional average TFR; the average number of children born per woman is 1.6 in the EU (35). Evidence shows that one of the reasons is people choosing to delay having children. In the current economic and social environment, women around the world are giving birth later in life; in HICs the average age at first birth is 30 for women and 33 for men (36). Due to the inverse relationship between age and fertility, this means that that people are likely to have fewer children than they planned. However, according to evidence from recent literature and the Policy Forum experts, there needs to be more education about how to promote and preserve fertility (37).

The decision to have children at a later age and a lack of infertility awareness also reduce the chances of treatment success. The Society for Assisted Reproductive Technology (SART) data show that the probability of getting pregnant with ART is significantly reduced with older age while the probability of pregnancy loss after undergoing ART treatment increases; a 2021 study reports that for women age 43 or older, the likelihood of pregnancy loss is 62% (38, 39). Therefore, delaying having children can have significant implications on family planning outcomes.

Although these trends are also influenced by changing social norms and lifestyle choices, they are underpinned by a lack of investment in public education about fertility and infertility. For instance, evidence from Italy demonstrates limited awareness of age-related decline in fertility among female university students. Similarly, a global systematic literature review concluded that university students have low awareness of fertility and lacked sufficient knowledge on fertility issues. Currently, sexual and reproduction education at school typically focuses on pregnancy prevention and does not cover infertility and fertility care in depth or at all, resulting in many seeking medical help for infertility too late because they are unaware of the risks of delay (40). Young people and their parents need better awareness and education regarding prevention of infertility through avoiding sexually transmitted diseases and unwanted pregnancies, and the positive impact of healthy lifestyles with respect to diet, exercise, weight, smoking, alcohol, use of drugs and avoidance of environmental toxicants (41). Furthermore, in some communities, there is still a stigma around infertility and its treatment methods, particularly ART (42, 43).

#### Lack of support for fertility preservation

The public's and policymakers' lack of awareness of infertility as a disease and the impact of age has consequences, including lack of support for potentially effective infertility management strategies. For example, egg freezing can potentially reduce the consequences of infertility later in life in patients with diseases affecting their fertility potential. Initially successful in 1986, the technique remained infrequently utilised due to limited success rates, despite ongoing developments in cryopreservation methods (44). The introduction of vitrification significantly enhanced clinical outcomes, leading to its reclassification in 2012 as a standard, non-experimental practice (45). Since then, egg freezing has become increasingly important for women at higher risk of becoming infertile due to cancer treatment or fertility-threatening medical conditions. The latter include but are not limited to autoimmune disorders, BRCA1/2 carrier status, and severe endometriosis. Egg freezing can preserve the ability to conceive a genetically related child and provide assurance to patients. International and country-specific professional societies have published guidelines dedicated to medical egg freezing. In 2019, the American Society of Reproductive Medicine (ASRM) released a committee opinion document emphasizing the need for better access to fertility preservation options for patients about to undergo cancer care (46). Similarly, the European Society of Human Reproduction and Embryology (ESHRE), has published guidelines that highlight the importance of fertility counselling for cancer patients to facilitate early decision-making concerning fertility treatment options (47). However, without reimbursement, medical egg freezing may be prohibitively expensive for most; data from the US reports costs of up to 15,000 USD per egg freezing cycle, plus the cost of medications (3,000–8,500 USD), storage (200–1,800 USD), and other costs (48–50). Subsequently, an IVF cycle must be paid for to use the eggs.

There are alternative reasons to freeze eggs beyond immediate medical indications. Young women may be interested in egg freezing to delay pregnancy until they are economically, socially, and mentally prepared to have a child, at which point they may experience age-related infertility. Professional societies have published guidelines covering planned egg freezing (also called: elective and non-medical). In 2024, ASRM released an ethics committee opinion highlighting that due to the novelty of this treatment, uncertainties still exist about its efficacy and safety, specifically long-term effects for the embryo. Therefore, ASRM recommends extensive counselling prior to treatment initiation to ensure that patients are informed about such uncertainties (51). ESHRE guidelines emphasise that women should be informed that while egg freezing may offer an option to extend fertility, it does not guarantee future pregnancy (47).

Recently, the demand for planned egg freezing has increased dramatically; between 2010 and 2015 the number of egg-freezing cycles grew by approximately 300% in Australia and New Zealand and by 900% in the United States (52). Studies indicate that this spike in demand is primarily fuelled by patients seeking to delay pregnancy until a more appropriate time in their reproductive life (53).

However, some countries only reimburse egg freezing services for immediate medical reasons, and others offer no reimbursement regardless of indication. According to the International Federation of Fertility Societies (IFFS), only 35% of countries offer some form of reimbursement of medically indicated egg preservation (54). Even large economies with low TFRs may not be offering reimbursement, e.g., in Canada patients may have to pay between 10,000–14,000 CAD (approximately 7,300–10,200 USD) for egg freezing after receiving a diagnosis (54, 55). Alternatively, in countries where ART treatment is mostly privately provided, employers may cover egg freezing for their employees through benefits e.g., in the US, according to data from 2020, 19% of large companies (with 20,000 or more employees) cover planned egg freezing (56). However, this demonstrates the inequity of access to affordable planned egg freezing services.

According to experts from the Policy Forum, the lack of funding for medical egg freezing services is especially concerning because, for example, cancer treatments may be necessary where egg freezing is indicated and this may occur even in very young patients (57). Earlier diagnosis and treatment can lead to early loss of fertility for many, which leads to significant challenges later in life. Additionally, survival rates of cancer patients have improved, which results in a larger pool of patients seeking ART treatment (58).

### Access to ART treatment

#### Insufficient or inequitable access to fertility treatment centres

Across studies and geographies, it has been documented that patients face barriers when seeking access to ART treatment. The number of ART cycles performed across the globe and within regions is highly varied (59). Evidence from Europe shows that ART utilisation ranges from 907 ART cycles per million in Portugal to 3,008 cycles per million in Denmark (59). Based on health economic estimates, to meet the needs of approximately 1,500 couples experiencing current infertility and meeting standard indications for IVF/ICSI, an estimated two ART centres per million population would be necessary. Each centre would need to serve over 750 couples annually to adequately address this demand (60). If one considers ART utilisation as a proxy for patient access—as proposed by Dyer et al. (18)—variability in reported ART cycles points to the existence of barriers to treatment.

The reasons behind limited patient access to infertility treatment differ by region, with some stemming from political factors while resource allocation decisions fuel others (61). However, a primary hurdle is the availability of appropriate services—the existence and accessibility of ART clinics. Access to treatment is often considered from an economic and legislative point of view, but the geographical dimension is paramount in countries with a well-defined urban/rural divide (62). An example is Brazil, a large country with a few urban clusters and a multitude of rural settings, in which patients lack convenient access to ART treatment. Most ART treatments are provided in large centres in the south and southeastern regions of the country where the largest cities (Rio de Janeiro and Sao Paolo) are located; the northern regions are deprived of access (63). In such situations, rural patients must travel long distances for consultations, putting more financial and emotional strain on them. Successful outcomes from ART treatment require the utmost engagement from the patient and, therefore, are associated with multiple consultations, appointments, and procedures. Extensive travel is likely to increase economic, social, and emotional costs (64). To receive treatment, patients must take time off work, disconnect from their social community, and reserve accommodation.

Patients in countries with higher urbanisation may also experience access challenges, such as long waiting lists. For instance, in the United Kingdom (UK), the average waiting list for ART treatment can reach three years (61). In Northampton it takes on average 21 weeks to access NHS treatment after being referred by a general practitioner (GP); in other cities, such as Leicester, it is up to 73 weeks (65). As age is a vital driving factor of successful outcomes in ART treatment, such delays can be detrimental to patients.

#### Lack of public service provision and stringent reimbursement criteria

According to experts from the Policy Forum, public treatment reimbursement is a key determining factor of patient access to ART treatment. Studies have shown that having little to no public coverage acts as a significant barrier to treatment (62). The financial structure of a country's fertility funding scheme can therefore enable or impede patient access to care. Ultimately, two broad archetypes of ART treatment funding systems exist: a generally government-funded system, and one driven by out-of-pocket payment by patients.

**Figure 1:**
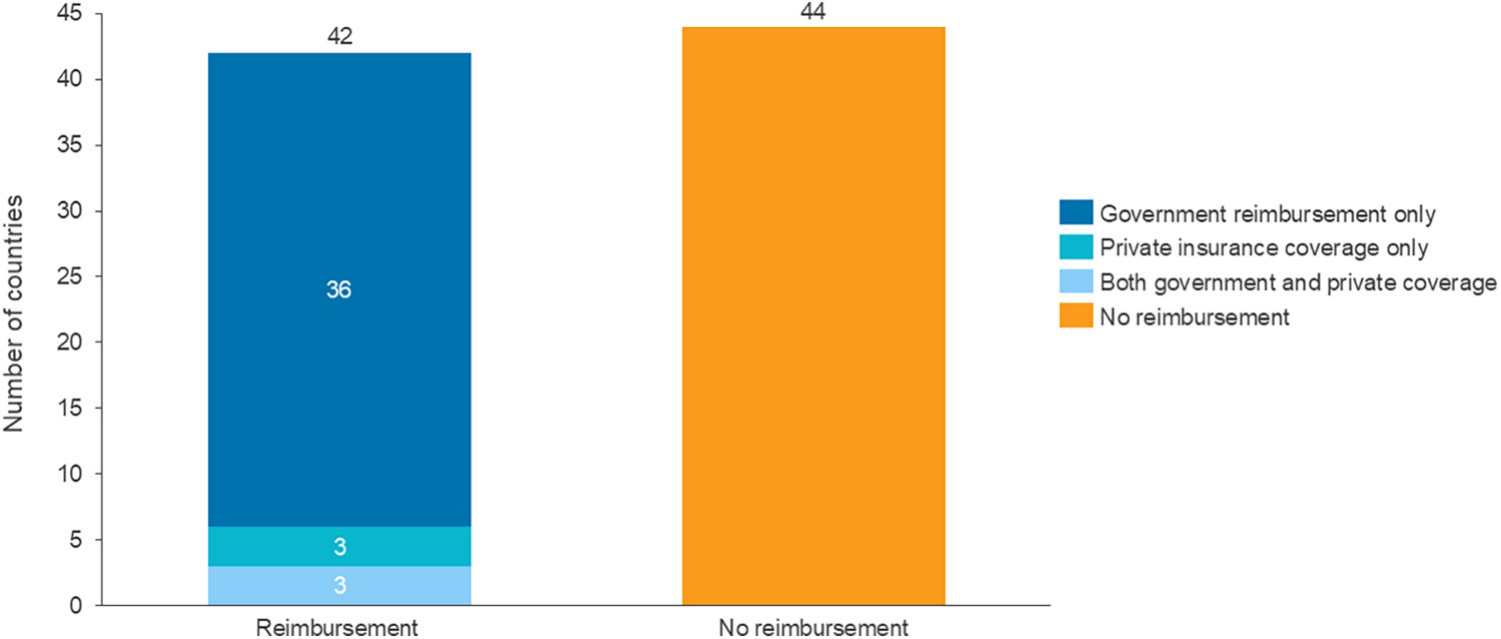
Reimbursement of ART treatment across 86 countries. Source: CRA analysis of IFFS Surveillance Report 2022 (54).

Countries with a fertility market driven by private care face inequity challenges and affordability concerns for patients because the cost of many ART treatments can be prohibitive. According to the IFFS, more than 50% of countries do not have any available funding for IVF or other types of ART treatments (Figure 1) (54). For instance, in Colombia, all fertility centres are private, and no insurance coverage is provided, so treatment is unavailable to most patients. Similarly, in the US, fertility care is primarily covered by private insurers; however, comprehensive coverage for IVF is mandated in only 13 states. Therefore, lack of reimbursement is a prominent access issue across regions (66).

The direct, negative effect that low or no government reimbursement of ART treatments has on the number of successful IVF cycles has been amply recorded in literature and pointed out by fertility experts (67). For example, in 2004, as a cost-saving measure influenced by the low prioritisation of infertility as a disease, the German government halved ART treatment reimbursement. A year after the introduction of the policy, Germany saw a 53% reduction in IVF cycles, demonstrating the short-term responsiveness of patient demand to a change in the cost of treatment (68). According to fertility experts, the increased cost of treatment borne by the patient may result in postponement: patients will wait longer before going to an ART centre as they will not feel financially prepared. Some may continue attempting pregnancy without medical intervention, without establishing the probability of success. Because infertility is a highly age-sensitive disease, this exacerbates low fertility rates and worsens patient outcomes, potentially reducing the cost-effectiveness of treatment that patients do eventually receive.

Where public funding is available for infertility care, reimbursed services typically include IVF and IUI. However, experts in the Policy Forum highlighted that the level of reimbursement differs significantly across countries as well as patient types. Different types of reimbursement limitations exist. The following are examples (69):

• **Age**: Maximum female or male age; treatment may be reimbursed only for women of a certain age. For in instance, in some regions of the UK only women up to the age of 35 can receive reimbursement (70).
• **Body mass index (BMI)**: A patient must have a (body mass index) BMI between 19 and 30 to qualify for IVF reimbursement in the UK and Ireland (70, 71).
• **Number of treatment cycles**: A limited number of IVF cycles may be reimbursed, after which the patient must fund any remaining treatment privately.
• **Marital status**: Reimbursement may be offered only for heterosexual, married couples.
• **Bureaucracy**: Only patients who can obtain proof of their infertility may receive treatment, e.g., they have been trying to get pregnant for over one year or have proof of a medically indicated reason for infertility (which may be difficult or impossible to demonstrate clinically).
• **Number of children**: Funding may be provided only to people with no other living children, irrespective of the present reason for infertility.

All or some of these factors may decide whether the patient is eligible for government reimbursement. Stringent reimbursement criteria may create inequity challenges for patients who do not meet them and push them into the private sector, exacerbating affordability concerns. Age restrictions for treatment reimbursement provide unique challenges, as women are reportedly planning to have children later in life, the limited funding lowers probability of conception. For instance, unmarried women are becoming a sizeable share of patients seeking IVF treatment. In the UK, Human Fertilisation and Embryology Authority (HFEA) data have shown that the number of IVF cycles and donor insemination treatments in unmarried women increased by over 100% between 2008 and 2020, and by 44% between 2019 and 2021 alone (72). In the many countries that refuse reimbursement to such a large group this may have a negative effect on the national fertility rate as many of these patients will be unable to afford private treatment.

An example of a country with complex restrictions is Italy, where couples, before being classified as eligible for partial reimbursement of ART treatments, must present a certificate of infertility that notes the clinical reason for infertility (73, 74). Spain has a similar policy. This raises a hurdle for patients, as the reason for infertility is often difficult to establish, and multiple tests must be conducted to diagnose an abnormality that may be causing the condition (73). Consequently, their access to treatment will be significantly delayed. Alternatively, they may find solutions outside their country and resort to cross-border care.

While it is reasonably expected that payers will apply some criteria to ensure cost-effectiveness of publicly reimbursed services, it is important to consider each patient's unique needs. Additionally, there are examples of economies that have successfully improved IVF outcomes while having more flexible reimbursement and subsidisation criteria, e.g., Taiwan, where the number of existing children is omitted from the reimbursement criteria (75). The 2021 expansion of the subsidisation criteria in Taiwan led to the birth of 20,539 babies through IVF by 2024 (76). This illustrates how more extensive funding can support successful outcomes for patients.

### Access to psychosocial support

Experts from the Policy Forum agreed that ART treatment has a significant impact on patients, both physically and mentally. Due to the stigma that still surrounds infertility, it can be stressful for patients to accept their disease, pursue a long treatment journey (seven-and-a-half months on average for IVF), and accept potential treatment failures (77). Therefore, ART treatment patients are at high risk of developing emotional disorders such as depression and anxiety or experiencing related symptoms (78). Studies have shown that 24% to 50% of infertile patients may display emotional health symptoms (79). Depression, anxiety, and distress may decrease a patient's fertility and thereby become a major driver of discontinuation of infertility care as patients become disincentivised by failed cycles of treatment; 20% of evaluated US couples who dropped out of treatment cited emotional distress as the primary reason (80).

The policy challenge associated with addressing patients' emotional distress is twofold. First, although international medical societies such as ESHRE have issued guidelines on the effective implementation of psychosocial care in fertility practices, many countries do not have psychosocial care as part of their fertility clinical guidelines, nor do they offer it for patients in public care (81). One example is Denmark, where national reimbursement of ART treatment is relatively high. However, according to experts in the Policy Forum, counselling is not offered or reimbursed for infertility patients, even though studies have indicated high levels of demand for it. In this case, financially well-off patients may self-refer to therapists who may not have experience with the issue of infertility, leading to further unresolved stress.

Second, some form of psychosocial care is reimbursed and offered in some countries, but patient uptake and engagement issues persist. According to experts from the Policy Forum, a lack of patient engagement can often result in ART treatment cessation. One example is the UK, where the National Institute for Health and Care Excellence (NICE) has issued official recommendations for all fertility centres to provide psychosocial support before, during, and after treatment; the HFEA also mandates this (82). Nonetheless, experts have pointed out that this recommendation is vague, as it suggests the centres “offer” counselling but may not ensure that it is sufficiently available for all patients. Whilst NICE issues national recommendations, regional integrated care boards are responsible for the level of reimbursement in their respective areas. Many clinics offer some complimentary sessions (usually one to two), but the rest must be paid for out-of-pocket by the patient. According to experts, due to the financial burden of payment and the additional stigma around mental health, many patients choose to forgo counselling.

### The use of supplementary care

Supplementary care refers to additional treatments to ART, such as preimplantation genetic testing (PGT) and add-ons, i.e., non-validated treatments that claim to improve ART treatment outcomes.

#### Variable access to preimplantation genetic testing

According to the committee opinion of the American College of Obstetricians and Gynaecologists, PGT “…comprises a group of genetic assays used to evaluate embryos before transfer to the uterus” (83). This encompasses PGT for monogenic or single-gene disorders (PGT-M), and PGT for chromosomal structural rearrangements (PGT-SR) (84). Previously, PGT was successfully used only for individuals with a known genetic disease who turned to IVF to prevent the child from inheriting it. However, new ways to use this technology as part of regular ART treatments have now been widely introduced despite conflicting with the recommendations by professional societies such as ESHRE and regulatory bodies such as HFEA (85).

Most professional and medical guidelines include recommendations and guidelines on best practices as they relate to PGT (86). Some countries do not have any strict regulations of PGT and instead leave the decision up to treating healthcare professionals (86). While this allows for flexibility around the provision of PGT, it also allows—in some cases—misinformed patient demand to drive supply. According to experts in the Policy Forum, in countries where various types of PGT are offered freely, some patients may go through numerous cycles of IVF attempting to conceive a child with specific traits, which raises ethical concerns.

Alternatively, some countries have taken a more structured and regulation-focused approach. In Germany, PGT is heavily regulated under the Preimplantation Act, which was instated in 2011 (87). PGT can be carried out only in specific genetic institutes and clinics and only after approval from the regional ethics committee. The Preimplantation Act 2011 was an update of the Embryo Protection Act of 1990, which prohibited PGT entirely, and substantial regulations around the procedure still exist. The restrictive nature of such laws can severely limit patient access, preventing them from pursuing further treatment.

Overall, both approaches to regulating and monitoring PGT can be associated with various challenges that increase barriers to optimal treatment access.

#### Use of non-validated treatments as “add-ons”

Non-validated treatments or “add-ons” are optional treatments which often come with claims that they can improve fertility outcomes. However, they may lack robust clinical evidence to support this. Because no high-quality, robust clinical trials confirm such treatments' value, their efficacy and safety profiles are unknown (88). For instance, assisted hatching is a procedure which is claimed to increase the chances of embryo implantation; however, medical societies such as ESHRE and governmental bodies such as HFEA have stated that there is insufficient high-quality evidence to support the procedure's efficacy (89, 90). Another example is endometrial receptivity testing, a test which is meant to determine the optimal time for embryo implantation, but some studies suggest that this procedure may reduce ART treatment effectiveness (90).

Additionally, PGT for aneuploidy (PGT-A), is considered a non-validated add-on although its value to patients is still being investigated (91). While PGT-M and PGT-SR (as described above) are usually offered as treatment options for individuals with presumed normal fertility but a known genetic disease of chromosomal abnormality, PGT-A is used for selecting euploid embryos, in the hope of increasing the live birth rate (92). While evidence on the impact of PGT-A on birth rates remains limited, the HFEA states that there is evidence suggesting it can reduce miscarriage rates (85). Professional, medical, and regulatory guidelines advise caution and additional careful consideration before using this technique as its efficacy is still undocumented [e.g., Canadian Fertility and Andrology Society (CFAS) (93), ESHRE (94), HFEA (85)].

According to experts, the provision of add-on treatments is driven by both demand and supply. From one perspective, patients often search for information on fertility treatments online, which may lead them to poorly validated success stories on social media featuring similar patients who were able to conceive with the help of a supplementary treatment. This pushes patients to seek out these treatments in private clinics with the hope of increasing their chances of fertility success, creating unrealistic expectations about their treatment outcomes and causing them to spend money that might be better spent on funding more IVF cycles.

From the supply side, according to the Policy Forum, some private fertility centres are incentivised to provide add-on services due to high demand and high potential profits. For example, in the UK, the cost of an add-on treatment such as assisted hatching may range from 130 GBP to 600 GBP (88). Without regulation, some clinics may advertise add-ons without providing robust clinical information about their efficacy and safety. A recent study has shown that of 254 reviewed infertility clinic websites, almost 80% provided an accurate description of the offered add-on procedures, but only 12% mentioned the lack of evidence that they are effective. Most importantly, none of the websites listed pregnancy rates following the add-on treatments (95).

In most cases, these add-on treatments involve an additional cost on top of the fertility treatment, to be paid out-of-pocket by the patient, which exacerbates affordability challenges for potentially misinformed patients. Supplementary treatments are often weakly regulated, leaving individual ART centres and providers as decision-makers. ESHRE has highlighted efficacy and safety issues regarding some add-on treatments and urges that all treatments offered be thoroughly analysed for their efficacy, safety, cost-effectiveness, and relevance before they reach patients (94). The key issue of providing and advertising add-on treatments is that even if the supply is motivated by patient demand, some add-ons may harm the patient, which introduces ethical concerns (96).

### Cultural, social, and religious considerations

#### Restrictive legislation based on marital status, same-sex, and single-parenting policies

The extent of access to ART treatments dramatically depends on a country or region's cultural, social, and religious context. Many countries adhere to the traditional view of a family: a married man and woman and their child who is genetically related to both. Access to ART treatment is therefore often restricted in such countries to married, heterosexual couples, thereby excluding single women and unmarried and non-heterosexual couples. The main guiding principle behind such restrictions is the welfare of the future child and concerns that it will end up in an “incomplete” and nontraditional family (97). It is necessary to recognise that such challenges are typically religiously and culturally motivated, rather than relating to economic considerations or policy priorities, and thus fall outside of the scope of this paper.

However, it is also important to note the stance of the WHO on this matter: all people should have access to health care without discrimination, regardless of their sexual orientation (98). The trends in family composition (and consequently the composition of the infertility patient population) are also changing, and thus existing policies are unsuitable for newly emergent segments of the population, including same-sex couples, unmarried couples, and single women. For example, in the UK, between 2019 and 2021, the number of infertility patients in female same-sex partnerships increased by 33%, while the number of single patients increased by 44% (72). This is further fuelled by the general global trend of postponement of parenthood by women choosing to further their career development before becoming a parent. However, in countries with more conservative attitudes towards single mothers, same-sex couples and marriage, these values are typically reflected in ART legislation, according to experts in the Policy Forum. Consequently, many patients seeking ART treatment face significant access hurdles.

#### Restrictive legislation on gamete donation and gestational surrogacy

Gamete donation involves the donation of eggs or sperm from fertile donors. Gamete donation is an option that enables people wanting a child, but who are unable because their own eggs and/or sperm are not capable of creating a pregnancy, to become parents. Services of donors are often sought out by cancer patients for whom egg freezing was not an option, same-sex couples, and those with medical conditions rendering their gametes inappropriate for ART treatments (99). However, due to cultural, social, and/or religious reasons, many countries, such as Germany, prohibit the donation of gametes, making it impossible for these patient groups to take advantage of fertility opportunities (100).

Gestational surrogacy is a service used when patients are not able to carry a child for medical or other reasons. This affects same-sex male couples and women who are unable to carry a child; for example, a woman with an absent or malformed uterus, recurrent pregnancy loss, or repeated IVF failures could benefit from seeking a surrogate (101). In some countries, the gestational carrier can be reimbursed for her expenses while pregnant or even be paid for her services. The latter is commercial gestational surrogacy and is allowed in only a few countries, including Ukraine, Mexico, and some parts of the US (102). At the other end of the spectrum, some countries, including France, Italy, and Germany, prohibit all forms of gestational surrogacy (102). Prohibition of gestational surrogacy, even based on cultural, social, or religious grounds, presents a challenge for many patients who are incapable of carrying a child for physical or medical reasons. With the growing demand for gestational surrogacy, challenges around access are becoming more prominent.

#### Summary of challenges

In conclusion, the policy challenges pertinent to the fertility space are multifaceted and differ greatly based on geography. Experts noted that the lack of patient access to ART treatment and optimal care remains a global concern. Diagnosing and addressing these challenges on a country-by-country basis would have a significant impact on patients' ability to access high-quality care and therefore could positively impact global fertility rates.

**Figure 2:**
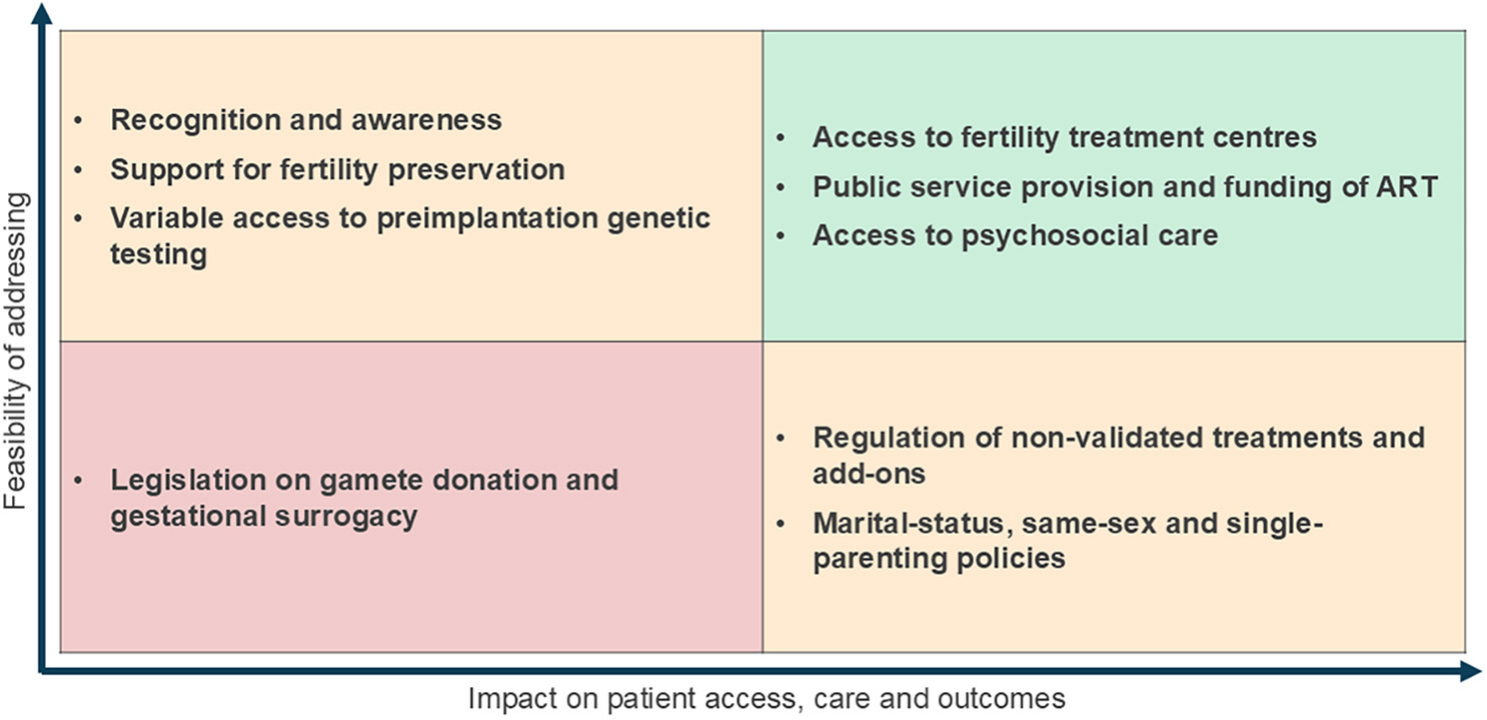
Global assessment of impact and feasibility of fertility policy challenges.

During the Policy Forum, the participants were prompted to categorise the discussed challenges based on their impact on patient access and the feasibility of addressing them. This allows us to see which challenges are the most impactful to patients and the most practical to address, i.e., those that should be prioritised for policy intervention. The framework below (Figure 2) provides a high-level overview of the discussion and its outcomes. Similarly, this framework may be used on a country level to assess the most pertinent challenges locally.

Addressing these challenges will be difficult for policymakers, as each challenge is associated with country-specific intricacies and drivers that must be considered when developing policies. For instance, factors such as the country's TFR, its level of economic development, the structure of its health system, and the cultural and religious context may greatly influence national policy priorities.

## Current advancements and innovative policy solutions

Several countries have implemented policies that have proven to be successful in addressing some of the challenges outlined above. We reviewed existing examples of policy developments to highlight best practices. Best practices represent examples of policies that have identified and aimed to address the fertility policy challenges discussed in “Challenges in accessing ART treatment and fertility care” section.

### Best practices to address recognition and awareness challenges

Experts in the Policy Forum agreed that fertility education needs to be at the top of the agenda of policymakers to make a major impact in preventing infertility and ensuring that patients seek fertility treatment (103). For example, Australia has taken steps to address this challenge effectively. The Australian organisation Your Fertility—which is funded by the Australian Government Department of Health and the Victorian Government Department of Health—has developed a national public health education programme. The programme aims to educate the general population on fertility and how a person can try to improve their chance of pregnancy and having a healthy baby (104). This programme addresses the challenge of a lack of recognition and awareness of infertility.

At an international level, it is valuable to emphasise the efforts of organisations. In 2023, the IFFS launched the More Joy campaign and toolkit which includes recommendations for policymakers to improve access to ART treatments (8, 105).

### Best practices for access to treatment

At an international level, various initiatives are monitoring the extent of access to ART treatment across countries, including the ICMART, IFFS, and European IVF Monitoring Consortium. However, it is the responsibility of national governments and payers to introduce policies to improve access to ART locally, and decision-makers in some countries have adopted forward-looking approaches to achieve this.

For instance, in Denmark, the cost of three fresh IVF transfers or five started cycles is funded by the public health service, provided that female patients are between the ages of 18 and 40. Since 2018, a new, equitable law enables heterosexual couples, single women, and lesbian couples to access infertility treatment. Furthermore, this policy has shown evidence of success as it is estimated that one in eight of all births in Denmark involve ART (9, 106, 107). There have also been more recent developments; starting December 1st of 2024, the government will provide individuals with free fertility treatment for a second child and increase the maximum number of fresh IVF cycles from three to six (108).

Access policies can also have a greater impact when implemented in combination with other types of support. For example, since 2022, Japanese public health insurance has reimbursed 70% of the costs of ART (although coverage excludes procedures such as genetic screening and the use of donor eggs) (109). In addition, new legislation has been introduced to allow national public employees up to ten days of paid leave a year to receive fertility treatment, addressing the fact that 62.5% of people undergoing IVF in Japan say it is very difficult to balance it with work; 11.3% say it is near impossible (110). In conjunction, these two polices aim to address the challenge in accessing treatment holistically, as in addition to the financial constraints they take into account the difficulties that patients face in balancing fertility treatment with work. The success of these policies could be measured through the percentage of newborns conceived through IVF, which in 2019 was estimated to be one in 14 (109). It is important to note that pro-natalist policies focused only on the social aspect have been shown to have a limited impact on birth rates (111, 112).

Finally, in the UK, the NHS funds fertility preservation for patients diagnosed with cancer. For example, Guy's and St Thomas’ NHS Foundation Trust also offers innovative ovarian tissue cryopreservation services to women undergoing cancer treatment. This provides cancer patients with opportunities to have biological children in the future and reflects societal desires for healthcare professionals to support cancer patients in preserving their fertility. Such policies are helpful examples of how high-cost funding interventions can be implemented in stages, to observe their effectiveness before more widespread policies are established. In this case, funding was provided to patients within a narrower age bracket and then expanded, with the latest policy offering young women aged 14 and over who are going through chemotherapy (113).

### Best practices in improving access to psychosocial support

Experts in the Policy Forum agreed that the psychosocial support provided to patients undergoing ART treatment is inadequate in many countries, and this impacts treatment outcomes because some patients discontinue treatment due to its psychosocial toll. There was consensus among the fertility experts within the Policy Forum that in countries where ART treatment is partially or fully publicly funded, psychosocial sessions should also be reimbursed or at least partially subsidised by the government. In private clinics, experts propose that the cost of these sessions needs to be incorporated into the service. Some existing policies aimed at supporting patients throughout their treatment journey can be looked to for lessons.

In Victoria, Australia, fertility counselling is a mandatory component of the Assisted Reproductive Treatment Act 2008 and the Assisted Reproductive Treatment Regulations 2019. All accredited clinics must offer counselling to individuals undergoing IVF and related procedures. Counselling is offered free of charge within the public fertility care system to eligible Medicare card holders, while private clinics may charge fees depending on their billing practices (114). Such psychosocial support helps patients develop strategies for different scenarios, including preparing for ART treatment and making decisions about the treatment; coping with unsuccessful treatment cycles or pregnancy losses; addressing specific concerns related to donor treatment cycles or feelings of anxiety or loss of control (115).

### Best practices in the use of supplementary care

A range of approaches have been utilised to guide and manage the appropriate use of PGT-SR/M, and to manage the use of non-validated “add-on” fertility treatments. For instance, in Australia, since November 2021, patients who meet a set of criteria have been able to claim a Medicare rebate for several PGT services, including PGT-M (monogenic) for couples at risk of passing on recessive, autosomal dominant, or mitochondrial disorders; PGT-SR (structural rearrangements) for carriers of chromosomal rearrangements; and PGT for sex selection for couples at risk of passing on X-linked disorders (116).

Similar policies are in place in Spain. PGT is available to various patient groups, including couples having ART who are at risk of chromosomal abnormalities, women aged 35 and over, couples with a history of chromosomal problems, and couples who have had repeated miscarriages (117). This policy has facilitated access to PGT for patient subgroups with an underlying clinical rationale.

The UK has been highlighted as a best practice in terms of add-on treatment regulation. The UK's HFEA has developed traffic-light ratings for supplementary care that is claimed to improve the chances of having a baby (live birth rate) but for which supportive evidence for most fertility patients is missing or not very reliable. The ratings are decided by HFEA's Scientific and Clinical Advances Advisory Committee subcommittee, which every 12 months reviews the available research for each treatment add-on in its traffic-light-rated list to determine whether the evidence base has changed (90). In general, HFEA's traffic-light-ratings framework helps inform patients wishing to use “add-ons” and aims to expand its scope as more of such treatments emerge. The organisation invites individuals to request that treatments of interest be added to its list. A limitation of this framework is that the evidence is reviewed only every 12 months and thus may hinder the uptake of innovative treatments or existing treatments for which new evidence is available (63).

### Best practices for cultural, social, and religious considerations

Various best practices exist for countries and regions advancing local legislation to reflect a country or region's cultural norms. Below we described two examples, from Australia and Denmark, that demonstrate how policies have been designed to address cultural and social concerns around fertility care.

In Australia, a person born from donor gametes is entitled to know the identity of their donor, and to meet with them, should they want this information once they turn 18. Therefore, donors must consent to their identifying information being held by the IVF clinic and also on a State registry. Western Australia, New South Wales, and Victoria have donor registries which allow children born from donor gametes to access their records beginning at 16 years old. The information includes all medical and family history, identifying information about the gamete donor, and the number and gender of persons conceived using the gametes provided by the same gamete donor (118). This legislation addresses the questions that people born from donor gametes may have about their genetic origins.

In 2018, the Danish parliament legalised double donation (both the egg and sperm cells come from donors) (119). Before 2018, Danish law stipulated that a child must be genetically linked to at least one of its parents via either their mother's egg or father's sperm. This presented a problem for three groups: heterosexual couples who both suffered from fertility issues, single women with poor egg quality, and lesbian couples in which the designated birth mother could not conceive using her own eggs (120). The updated legislation reflects the generally liberal views of Danes and addresses concerns raised by patient groups about Danish women travelling abroad for treatment with double donation (120).

## Discussion: goals for optimising patient access and care

Building from the above evidence of challenges to optimal ART treatment and examples of best-practice policies that exist across different regions, this section covers potential policy goals as suggested by the panel of fertility experts involved in the Policy Forum. Specifically, we will discuss their importance, their geographical relevance, and the approximate timeline required to implement them. Country-specific tailored recommendations are outside the scope of this paper and should be the object of further research. For instance, countries in the Asia Pacific region have the highest rates of infertility globally, and hence warrant additional targeted research and goal setting (2).

In this paper, we aim to offer high-level global goals to support and guide the direction of local policymaking. Further research should focus on definition of country- and healthcare system-specific objectives and strategies. Therefore, the following section should be interpreted as a general guide rather than formal policy recommendations.

### Goals for improving recognition and awareness

Experts reached a consensus on the fact that raising awareness about infertility and how to mitigate it is paramount. Consequently, they proposed that increasing political recognition and establishing education campaigns for different demographic groups is an actionable goal that should be undertaken globally in the short to medium term.

While the WHO has recognised infertility as a disease, additional efforts are required to align local decision-makers with this definition. This is paramount for improving patient access and helping resolve the growing infertility crisis. Recognition and regulation of infertility as a disease could set the path for the resolution of challenges related to the availability and affordability of ART treatment by potentially enabling legislative change and increasing the proportion of the healthcare budget allocated to tackling infertility. Nonetheless, the extent of action will inevitably vary based on geographic region and level of economic development; some countries, particularly LMICs, may have too many competing health priorities to substantially increase the allocation of the share of their finite healthcare budget that goes to infertility. However, since the right to found a family is a fundamental human right, infertility should be considered in an equitable manner with other societal medical needs.

Policymakers, together with the stakeholders involved in providing ART treatment, can develop national infertility plans to set evidence-based goals for fertility-specific policies, treatment, and care. This would ensure that the specific fertility challenges observed locally are adequately addressed through the national plan. Additionally, national policymakers can seek the support of international organisations when drafting such plans e.g., the More Joy Toolkit developed by the IFFS (8). These plans will support local stakeholders in having a unified vision and working towards the same goals.

Experts in the Forum highlighted the importance of investment in awareness campaigns to avoid exacerbating the trends of (and problems associated with) ageing populations and postponement of childbearing. Based on their geographic region and local disease aetiology, patients need to be informed about the factors that could affect their fertility. That will give patients the opportunity to have more control over their fertility. Moreover, different demographic groups, including adolescents and adults, must be covered within targeted education campaigns. Such educational campaigns should inform individuals facing fertility challenges but who have not yet accessed medical support of the treatment options available to encourage them to seek medical advice within an appropriate time frame.

Governments should increase funding to fertility preservation for patients diagnosed with cancer or other conditions that compromise fertility to ensure that such patients have access to fertility services. Oncofertility-specific funding will help alleviate the stress cancer patients face throughout their treatment and give them the opportunity to achieve their family goals.

To conclude, Table 2 provides a summary of the covered fertility policy goals to improve the recognition and awareness of fertility.

**Table 2:** Fertility policy goals to improve the recognition and awareness of fertility.

Fertility policy goal	Geographical relevance	Timeline
Recognise infertility as a disease by key national stakeholders (e.g., policymakers and payers) and society	Global	Short to medium term
Prioritise infertility as a disease like any other within healthcare systems (e.g., by establishing and utilising medical codes for all fertility procedures)	Global	Short to medium term
Develop national plans on infertility covering fertility specific policies, treatment and care, and the stakeholders involved in providing an organised service	Global	Medium to long term
Establish widespread education campaigns, addressing different age groups, to improve awareness of infertility and fertility	Global	Short term
Establish targeted information campaigns on available ART options for individuals experiencing infertility	Global	Short term
Increase funding and access to fertility preservation for patients diagnosed with cancer or other conditions that compromise fertility	Global	Medium term

Source: CRA analysis and input received during the expert Policy Forum.

### Goals to improve access to ART treatment

Patients around the globe are facing various challenges in accessing ART treatment, primarily due to a lack of public and private service provision and restrictive reimbursement criteria. There was consensus among fertility experts that international stakeholders should set ambitious targets and aim for global access to ART to at least double within a decade.

National- and regional-level stakeholders need to find local solutions to meet this global goal. For example, depending on the country's healthcare system framework, this goal could be achieved in the following ways:

• Establishing or increasing the proportion of the healthcare budget that is allocated to tackling infertility
• Scaling up public service provision and availability to reduce wait times at public ART clinics
• Introducing affordability schemes for patients with the least ability to pay to tackle access inequities
• Expanding reimbursement criteria to ensure that they reflect international medical standards for optimal access and care (regarding which patients can access treatment and for how many cycles)
• Leveraging advanced digital tools such as artificial intelligence (AI) to optimise ART treatment delivery through workflow optimisation

By lifting the financial burden of the disease from patients, policymakers will make treatment more accessible for a larger pool of patients and potentially help increase local fertility rates.

Recognising that the underlying access challenge varies significantly across countries, experts in the Policy Forum developed a set of goals to reflect the need to first diagnose what is currently failing in the access environment and thus inform policy interventions that will address these underlying failures (Table 3).

**Table 3:** Fertility policy goals to improve access to fertility treatment.

Fertility policy goal	Geographical relevance	Timeline
Conduct robust assessments of the prevalence of infertility at a national level and assess the ability of fertility services to meet this demand	Global	Short term
Diagnose access environments for ART treatment at a local level by establishing rates of access (e.g., number of cycles, wait times, out-of-pocket costs) and identifying factors contributing to low or inequitable access	Global	Short term
Adopt tailored solutions to increase ART access for patients (e.g., by introducing affordability schemes, increasing government funding, expanding reimbursement eligibility)	Global	Medium term
Double global access to ART within a decade	Global	Medium to long term

Source: CRA analysis and input received during the expert Policy Forum.

### Goals to improve access to psychosocial support

Across most healthcare systems, general access to adequate mental health care is limited (121). This is especially true in regard to the psychosocial support provided to patients undergoing fertility treatments (122). Experts stressed that patient engagement determines the uptake of the provided support and, thus, also affects fertility outcomes. To limit cessation of treatment, patients must be offered psychosocial support before and during treatment, as it helps set realistic expectations.

There was consensus among the fertility experts during the Policy Forum that in the short to medium term, patients need to have better access to psychosocial care before and during treatment. This goal can be achieved by allocating targeted government funding and emphasising the importance of psychological support to providers and patients. While some countries already offer an introductory counselling session for patients, there needs to be more encouragement to engage in such sessions so they can see the benefits of psychosocial support.

Furthermore, patients can become better engaged with their treatment if they have frequent check-ins and digital support that can guide and support them throughout the treatment journey. This will allow patients to have more control over their care and ease into treatment on their own terms and timeline. Within the medium term, the availability of digital tools (e.g., online support, mood trackers) used to support patients undergoing fertility treatment needs to be monitored and funding allocated to support the use of effective tools. To be successfully integrated into the pathway, digital tools need to adhere to quality standards and be accessible by patients.

These goals are summarised in Table 4. The success of their implementation could be measured by the change in IVF cycle uptake across involved regions, given the link between treatment uptake and availability of support. However, it is important to recognise that, in some countries, these goals may be deprioritised due to more persistent access or affordability challenges; access to treatment is a more urgent priority and must be addressed before the improvement of psychosocial support to patients can take precedence. Therefore, the goals could be achievable in the short term in regions that already have reasonable access to ART treatment (e.g., public reimbursement of treatment and sufficient service availability). In countries where access to treatment is still a significant challenge (e.g., no public reimbursement, major affordability concerns), psychosocial support goals may be more reasonably prioritised for addressing in the medium to long term.

**Table 4:** Fertility policy goals to improve access to psychosocial support.

Fertility policy goal	Geographical relevance	Timeline
Expand the availability of psychosocial care before, during, and after ART treatment through counselling sessions	Region specific	Short to medium term
Increase government funding allocated to psychosocial care for patients undergoing fertility treatment	Region specific	Short to medium term
Expand patient engagement offerings through access to appropriate tools (e.g., mobile apps)	Region specific	Short to medium term
Allocate government funding to the reimbursement of digital tools to support patients undergoing fertility treatment	Region specific	Medium term
Ensure that all patients undergoing fertility treatment have access to psychosocial support across all regions	Global	Medium to long term

Source: CRA analysis and input received during the expert Policy Forum.

### Goals to regulate the use of supplementary care

Patients undergoing ART treatment often feel influenced to use “add-on” treatments at their own expense, but they are not made aware of those treatments' unproven efficacy. To curb the global use of “add-on” treatments—including over-the-counter medicines and alternative therapies—in the short term, there need to be education campaigns targeting patients and scientific communication targeting fertility healthcare professionals on the efficacy and safety of such treatments. Such campaigns will help tackle both the demand for “add-ons” and the supply of them at fertility clinics. Similar campaigns can be launched to educate patients and healthcare professionals about when PGT is clinically recommended. This will help to prevent patient requests for unnecessary PGT services.

In the short term, existing international guidelines from reputable organisations, such as ESHRE and ASRM, should be systematically used by healthcare professionals in fertility clinics (89). These guidelines feature information about the efficacy and safety of the “add-on” service and when it should be used. Importantly, these guidelines should be frequently renewed to avoid misinformation being publicised and so both patients and healthcare professionals can easily access them.

Furthermore, regulations prohibiting marketing campaigns and digital applications that convey misinformation on supplementary care and regulation of supplementary care can eliminate or reduce the use of non-validated “add-ons” in the medium to long term. Many clinic websites feature advertisements for supplementary treatments they offer but do not provide relevant information about the efficacy and safety of these treatments. To avoid misinforming patients, regulations must be implemented to prevent such marketing.

These goals are summarised in Table 5.

**Table 5:** Fertility policy goals to improve the use of supplementary care.

Fertility policy goal	Geographical relevance	Timeline
Information campaigns targeting patients and scientific communications targeting fertility healthcare professionals on the limited efficacy of “add-on” treatments; campaigns and communications should reflect the latest evidence as assessed by professional organisations like ESHRE and HFEA	Global	Short term
Education campaigns targeting patients and scientific communication targeting fertility healthcare professionals on the eligibility criteria for PGT, as defined by the relevant professional body such as ESHRE and HFEA	Global	Short term
Implementation of local professional society guidelines (e.g., ESHRE, HFEA) on the use of supplementary care in the clinical setting and the updating of such guidelines as new evidence emerges	Global	Short term
Regulation of marketing campaigns conducted by fertility clinics regarding non-validated add-ons (e.g., material posted on their websites or social profiles) to include the associated efficacy and safety profile	Region specific	Medium to long term
Regulation of digital applications that aim to support people using fertility and ART services	Region specific	Medium to long term
An enhanced and improved regulatory and research environment to assess the clinical utility, efficacy, and safety of novel treatments	Region specific	Medium to long term
Promulgation of regulations to make commercially available only the add-ons that have been properly validated through clinical assessment of efficacy and safety	Region specific	Medium to long term

Source: CRA analysis and input received during the expert Policy Forum.

### Goals to support cultural, social, and religious considerations

Goals related to cultural, social, and religious considerations should be formulated at a national level to align with the country's general sociocultural context. While we can discuss the challenges and barriers certain groups face and advocate for improved and equitable access for all, national stakeholders must define the most appropriate policy response locally. However, national stakeholders should aim to advance legislation on fertility treatment to reflect the country's evolving cultural, social, and religious considerations (Table 6).

**Table 6:** Fertility policy goals around cultural, social, and religious considerations.

Fertility policy goal	Geographical relevance	Timeline
Advanced legislation on fertility treatment to reflect national and regional evolving cultural, social and religious norms	Global	Variable

Source: CRA analysis and input received during the expert Policy Forum.

### The roles of stakeholders in achieving goals

For fertility goals to be realised, national, regional and international stakeholders will need to collaborate. Table 7 provides an overview of the potential roles of the stakeholders involved in making this a reality. The fertility space features an intricate conjunction of challenges: access, affordability, legislation, and ethics are involved. Consequently, stakeholders from different parts of the healthcare ecosystem need to work collaboratively to achieve the aforementioned goals.

**Table 7:** The roles of stakeholders in achieving the fertility policy goals.

Stakeholder	Role
Academic community	• Generate evidence to support informed policy decision-making and to guide clinical guidelines • Drive basic research in the fertility space
Clinical community	• Maintain and comply with up-to-date clinical guidelines on ART treatment and supplementary care • Contribute in academic research to advance innovation • Develop material for targeted education campaigns • Disseminate scientific communication targeting healthcare professionals • Strive for technology improvements to improve live-birth rates and drive down costs through optimisation • Support policymakers in the development and implementation of fertility policies
International organisations	• Encourage governments to address infertility, an important component of sexual and reproductive health and rights (as highlighted in the WHO report) (2) • Collate evidence to support informed policy decision-making • Develop reports that highlight international, regional, and national fertility policy gaps • Set fertility targets and goals that support improved access to ART treatment globally • Support policymakers in the development and implementation of fertility policies
Patient advocacy groups	• Raise awareness about infertility and existing treatments • Disseminate educational campaigns targeting patients and the general population • Drive policymakers to implement policies that address the nation's fertility policy gaps • Advocate for expansions in funding allocated to infertility and for broader reimbursement criteria
Policymakers	• Recognise infertility as a disease • Develop cohesive, forward-looking fertility policies and fertility national plans • Establish public information campaigns and improve school education • Increase funding allocated to ART treatments and services, including psychosocial support services and fertility preservation services for cancer patients • Increase funding allocated to fertility research
Regulators and legislators	• Recognise infertility as a disease • Regulate supplementary care and its marketing • Regulate digital tools used to support patients undergoing ART treatment • Advance legislation on ART treatment and update it to keep it up to date on latest developments
Pharmaceutical, digital and tech industries	• Inform policymakers about access challenges in fertility care • Inform policymakers, regulators, and legislators of upcoming innovation (for horizon-scanning exercises) • Recognise their role in facilitating access to sexual and reproductive health services • Develop and implement strategies to improve access to sexual and reproductive health services • Develop innovative products for ART treatment and to support patients throughout their patient journey

Source: CRA analysis and input received during the expert Policy Forum.

First, there needs to be strong collaboration between the academic community, clinical community and industry for innovative treatments to be developed, tested, commercialised and made available to patients. It is paramount for the academic and clinical communities to conduct the anticipated research and generate evidence to support the adoption of safe and effective innovative technologies and inform the development of fertility policies that consider upcoming innovations and developments in clinical practice. The government and the pharmaceutical industry could support the generation of such research and innovation through grant funding. With the emergence of new technologies, stakeholders can harness real word data to help inform research and treatment development.

Second, close collaboration between the clinical community, international organisations, patient advocacy groups and policymakers will ensure that the developed policies address the needs of patients and the nation's most pressing policy gaps. Many experts perceive that patient advocacy groups are often one of the strongest voices in renegotiating legislation and reimbursement provisions, as they have experienced these challenges firsthand. With the support of the clinical community, patient advocacy groups can better formulate the objectives and needs of patients and feed them into policymaking discussions. The involvement of the clinical community will also help highlight the medical impact of outlined goals, thereby evidencing the importance of achieving them. In order to support the affordability of fertility care along with expanding access to it, efficiency-maximising tools should be leveraged; existing evidence highlights that introduction of AI can play a role in reducing the cost of fertility care through workflow optimisation in fertility clinics and machine learning-supported mapping of personalised treatment protocols (123, 124). Another study highlighted the cost-saving potential of telemedicine, when used in fertility care, demonstrating that virtual consultations and remote monitoring can reduce the need for in-person visits, lower travel expenses for patients, and streamline clinical workflows (125). Such tools and initiatives can be mirrored to reduce the cost of fertility care provisions.

Furthermore, the industry can inform policymakers of any access challenges in fertility care, provide international lesson-sharing, and keep decision-makers updated on upcoming innovations.

To summarise, while stakeholders have different roles, it is paramount for policymakers to collaborate across the board to develop and enforce optimal fertility policies that maximise patient access.

## Conclusion

Infertility should be recognised as a serious medical condition and a priority for society. However, in many countries this is not the case. This paper is the result of collaboration by leading experts in the field and has resulted in ambitious and relevant policy goals being established. However, implementation will require efforts and action from various national, regional, and international stakeholders—covering the academic and clinical communities, patient advocacy groups, policymakers, regulators, legislators, and the industry—to ensure all patients have access to optimal fertility care. Policymakers should aim to address fertility care challenges holistically by ensuring availability of ART treatments and psychosocial care, and enabling adequate patient access to such services. Furthermore, frameworks need to be in place to ensure that patients utilise only efficacious and clinically recommended care. These fertility goals can be achieved only if all key stakeholders recognise infertility as a serious disease that requires investment to address the needs and rights of affected individuals and benefit society today and in the future.

## Footnotes

^1^Throughout this report, the term “women” does not exclude transgender persons.
